# Comparación diagnóstica de la ecografía pulmonar abreviada a pie de cama y la radiografía de tórax en la unidad de cuidados intensivos

**DOI:** 10.23938/ASSN.1088

**Published:** 2024-11-15

**Authors:** Juan-Ambrosio Martínez-Molina, Miguel Ángel Martínez-González, Marc Vives Santacana, Ariel Duilio Gonzalez Delgado, Karlos Reviejo Jaka, Pablo Monedero

**Affiliations:** 1 Policlínica Gipuzkoa Servicio de Anestesiología y Reanimación San Sebastián España; 2 Clínica Universidad de Navarra Departamento de Anestesiología y Cuidados Intensivos Pamplona España; 3 Universidad de Navarra Facultad de Medicina Departamento de Medicina Preventiva y Salud Pública Pamplona España; 4 Instituto de Investigación Sanitaria de Navarra (IdiSNA) Pamplona España; 5 Biomedical Research Network Center for Pathophysiology of Obesity and Nutrition (CIBEROBN) Carlos III Health Institute Madrid Spain; 6 Policlínica Gipuzkoa Servicio de Cuidados Intensivos San Sebastián España

**Keywords:** Ultrasonografía, Radiografía Torácica, Cuidados Críticos, Atelectasia Pulmonar, Ultrasonography, Radiography Thoracic, Critical Care, Pulmonary Atelectasis

## Abstract

**Fundamento:**

La ecografía pulmonar abreviada a-pie-de-cama (POCUS) ofrece ventajas respecto a la radiografía de tórax. Este estudio compara los hallazgos entre POCUS y radiografía portátil de tórax, así como el desempeño diagnóstico de la POCUS en la unidad de cuidados intensivos (UCI).

**Metodología:**

Se incluyeron pacientes adultos ingresados en la UCI. La POCUS se realizó utilizando el protocolo abreviado BLUE. Se compararon los hallazgos entre POCUS y radiografía portátil de tórax. El desempeño diagnóstico de la POCUS se analizó utilizando como referencia el diagnóstico clínico del intensivista, basado en la exploración clínica y la ecografía pulmonar, obteniéndose sensibilidad (S), especificidad (E), y valores predictivos positivo (VPP) y negativo (VPN).

**Resultados:**

Se incluyeron 100 pacientes, 71 con hallazgos de patología pulmonar. El tiempo medio para realizar la ecografía fue 308 segundos. La ecografía detectó patología en 20 pacientes con radiografía de tórax normal. Se observaron discrepancias diagnósticas en 30 pacientes, destacando la superior sensibilidad de la ecografía para detectar atelectasias, derrames pleurales y edema pulmonar. La ecografía mostró S=85%, E=100%, VPP=100% y VPN=55%.

**Conclusiones:**

La POCUS pulmonar al ingreso en la UCI detectó un mayor número de patologías y no omitió ninguna anomalía importante detectada en la radiografía. Además, mostró una buena exactitud diagnóstica. Estos resultados sugieren que la POCUS pulmonar, realizada con un protocolo abreviado, puede ser una alternativa viable a la radiografía de tórax en la evaluación inicial y el seguimiento de la patología pulmonary en pacientes críticos, impactando en la calidad de su atención y manejo.

## Abstract (en)

**Background:**

Bedside lung ultrasound (POCUS) offers advantages over chest X-ray, including better cost-effectiveness for diagnosing certain pulmonary pathologies. This study compares the dia-gnostic concordance between portable chest X-rays and bedside lung ultrasounds in the intensive care unit (ICU).

**Methods:**

Adult ICU patients were included. POCUS was performed using the abbreviated BLUE protocol. Diagnostic results from POCUS and chest radiographies were compared using the intensivist clinical diagnosis -based on clinical examinations and lung ultrasounds- as the reference. Sensitivity, specificity, positive predictive value (PPV), and negative predictive value (NPV) of the ultrasounds were calculated.

**Results:**

A total of 100 patients were included, 71 with pulmonary pathologies. The average time to perform the ultrasound was 308 seconds. Ultrasound identified pathology in 20 patients with a normal chest radiographs. Diagnostic discrepancies occurred in 30 patients, highlighting ultrasound´s superior sensitivity in detecting atelectasis, pleural effusions, and pulmonary edema. Ultrasound demonstrated sensitivity (S) of 85%, specificity (E) of 100%, positive predictive value (PPV) of 100%, and negative predictive value (NPV) of 55%.

**Conclusion:**

Lung point-of-care ultrasound at ICU admission detects more pathologies and does not miss significant abnormalities seen on chest X-rays. It also shows good diagnostic accuracy. These findings suggest that pulmonary POCUS, using an abbreviated protocol, could be a viable alternative to chest radiography for initial evaluation and follow-up of pulmonary pathologies in critically ill patients, potentially improving care quality and management.

## INTRODUCCIÓN

La tomografía computarizada de tórax ha sido tradicionalmente la prueba de imagen de referencia para la exploración del parénquima pulmonar. Sin embargo, su alto coste y los riesgos asociados, como la irradiación del paciente, la necesidad de transporte y la toxicidad del medio de contraste, han llevado a buscar alternativas en la evaluación pulmonar del paciente crítico^1^. En este contexto se ha utilizado comúnmente la radiografía de tórax portátil, pero su escaso valor clínico^2^^,^^3^ y las limitaciones técnicas a pie de cama han impulsado el interés en la ecografía pulmonar a pie de cama (POCUS)^4^ como medio de diagnóstico y seguimiento de la patología pulmonar del paciente crítico^5^.

La POCUS pulmonar ha experimentado un rápido desarrollo desde 2008, con la publicación del protocolo BLUE (*Bedside Lung Ultrasound in Emergency*)^6^, diseñado para reducir el tiempo de exploración ecográfica pulmonar en pacientes críticos con disnea. En 2012 se estableció el primer consenso internacional^7^ sobre el uso de la ecografía pulmonar, el cual ha sido actualizado recientemente^8^.

La POCUS pulmonar ofrece ventajas significativas, como la ausencia de radiación para el paciente y el personal, la facilidad de realización sin necesidad de transporte del paciente, y un mayor coste-efectividad en ciertas patologías pulmonares^9^^,^^10^. No obstante, su adopción como examen de rutina requiere que sea capaz de diagnosticar una amplia gama de patologías pulmonares con al menos la misma precisión que la radiografía de tórax, reduciendo así la necesidad de radiografías rutinarias en la UCI^10^^,^^11^.

El objetivo del presente estudio es evaluar la concordancia diagnóstica entre la ecografía pulmonar a pie de cama y la radiografía portátil de tórax realizadas al ingreso de un paciente en la UCI. La información obtenida permitirá aportar evidencia adicional sobre la utilidad diagnóstica de la POCUS pulmonar y su capacidad para reducir la realización de radiografías rutinarias en la UCI.

## MATERIAL Y METODOS

Realizamos un estudio de concordancia diagnóstica entre la radiografía de tórax portátil y la POCUS pulmonar con el protocolo BLUE, cuya recogida de datos tuvo lugar en las UCI de dos hospitales (Clínica Universidad de Navarra en Pamplona, y Policlínica Gipuzkoa en San Sebastián) entre septiembre de 2022 y enero de 2024. El estudio fue aprobado por el comité de ética de investigación de la Clínica Universidad de Navarra, que eximió del consentimiento informado de los pacientes por recoger datos de la actuación estándar.

Se incluyeron pacientes que ingresaron en la UCI tanto por motivos médicos como quirúrgicos, mayores de edad (18 años o más), a quienes se les realizó la exploración habitual mediante POCUS pulmonar y radiografía de tórax para evaluar la presencia de patología pulmonar. Se excluyeron pacientes sometidos a cirugía cardiaca y torácica debido a las limitaciones técnicas de las imágenes en estos casos, ya que la presencia de enfisema subcutáneo o grandes apósitos torácicos altera o impide la propagación de los haces de ultrasonido al parénquima pulmonar subpleural.

El estudio contó con la participación de tres médicos intensivistas expertos, instructores de cursos de ecografía cardiopulmonar, y tres residentes de último año con formación y con experiencia previa (al menos 40 exploraciones), supervisados por un experto en ecografía pulmonar.

**Figura 1:**
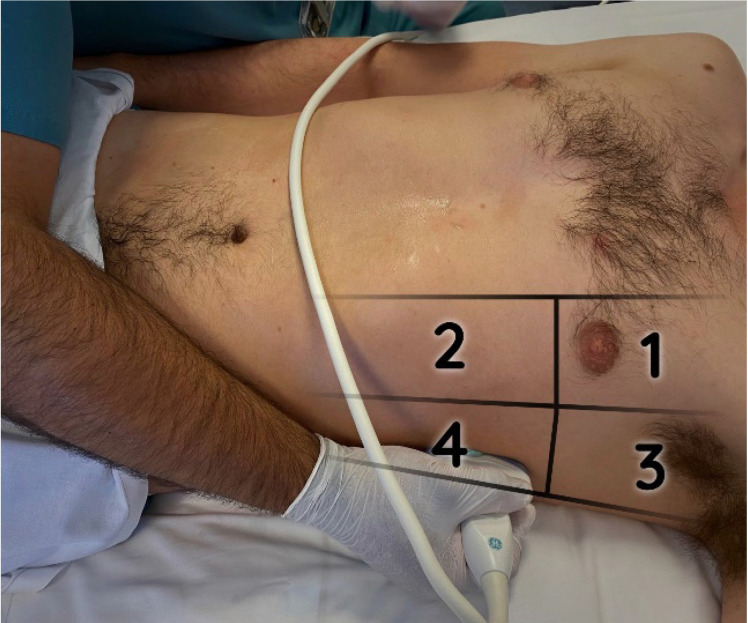
Puntos de exploración en la ecografía pulmonar a pie de cama. 1: anterosuperior, 2: anteroinferior, 3: posterolateral superior, 4: posterolateral inferior.

Se utilizó una versión abreviada adaptada del protocolo BLUE^6^^,^^12^, conforme a las recomendaciones internacionales para ecografía a pie de cama^7^. Se examinaron cuatro puntos pulmonares (Figura 1): dos anteriores (anterosuperior y anteroinferior) y dos posterolaterales (superior e inferior). Se evaluaron ambos pulmones en busca del signo del deslizamiento (*sliding*) pulmonar, presente en el pulmón normal e, indicativo de que las dos capas pleurales, parietal y visceral, están en aposición entre sí y se deslizan con la respiración. Los patrones ecográficos se clasificaron como A (normal), B (edema pulmonar) y C (atelectasia/neumonía), y se registró la presencia de derrame pleural y catéteres intravasculares.

Las radiografías de tórax se realizaron, como máximo, 60 minutos tras la realización de la POCUS pulmonar. Fueron interpretadas por el radiólogo asignado a la UCI, con más de 20 años de experiencia, y se establecieron los siguientes diagnósticos radiológicos: radiografía normal, aumento de densidad (atelectasia/neumonía), edema pulmonar, derrame pleural y presencia de catéter intravascular.

Se emplearon un ecógrafo móvil (Venue, GE Healthcare) con transductor curvilíneo y un aparato de radiología digital portátil (MobileDiagnost M50, Philips). Se cronometró el tiempo de la exploración ecográfica.

La comparación entre la POCUS pulmonar y el informe del radiólogo se realizó utilizando como patrón de referencia el diagnóstico clínico establecido por el intensivista. Este diagnóstico se basó en la valoración de los datos clínicos, la exploración física y las pruebas complementarias, incluyendo la ecografía pulmonar y la radiografía de tórax, analizadas por el intensivista antes de recibir el informe del radiólogo.

## Análisis estadístico

El tamaño muestral se calculó asumiendo una sensibilidad mínima del 75% (proporciones p =0,75 y q=0,25) para disponer de la capacidad de calcular un intervalo de confianza al 95% (z = 1,96) que tuviese una anchura a cada lado del 10% (M=0,1). Se aplicó la siguiente expresión^9^:

$$n=\;\frac{z_{\alpha /2}^{2}\;pq}{M^{2}}=72,03$$

Bajo estos supuestos, se determinó que se necesitaban 73 pacientes con ambas pruebas (radiología y ecografía) realizadas en cada uno. Se optó por una muestra de 100 sujetos para mejorar la precisión de los resultados y compensar posibles pérdidas de datos.

Se describieron las variables cuantitativas con media y desviación estándar (DE) y las cualitativas con frecuencia y porcentaje. El desempeño diagnóstico de la POCUS pulmonar se analizó mediante los parámetros sensibilidad, especificidad, valor predictivo positivo y valor predictivo negativo, junto con sus intervalos de confianza (IC) al 95%.

## RESULTADOS

Se incluyeron datos de 100 pacientes, 56 de la Clínica Universidad de Navarra y 44 de la Policlínica Gipuzkoa. Predominaron los varones (66%), con edad media 63,7 años (DE=1,3), peso 76,2 kg (DE=1,4) e índice de masa corporal 27 kg/m^2^ (DE=4,6). El 49% de los pacientes ingresaron por motivos médicos y el 51% por motivos quirúrgicos. El 57% fueron ingresos urgentes y el 43% ingresos quirúrgicos programados.

Tras el análisis de las radiografías de tórax, el radiólogo informó de aumento de densidad en 51 pacientes, describiéndolo como edema agudo de pulmón en 19 (37,3%) y como probable atelectasia/neumonía (a valorar clínicamente) en 32 (62,7%). Se informó la presencia de derrame pleural en cinco pacientes, sin que se observara ningún caso de neumotórax (Tabla 1).

**Tabla 1:** Comparación de hallazgos de ecografía abreviada pulmonar y radiología portátil de tórax

Diagnóstico de imagen	Ecografía abreviada n (%)	Radiografía portátil n (%)
Patrón A - Normal	29 (23,2)	49 (46,7)
Patrón B - Edema pulmonar	24 (19,2)	19 (18)
Patrón C - Atelectasia	38 (30,4)	32 (30,5)
Patrón C - Neumonía	18 (14,4)	
Derrame pleural	16 (12,8)	(4,8)

Respecto a la ecografia, el tiempo medio de exploración ecográfica fue 5 minutos y 8 segundos (308 s; DE=6,5; IC95%: 295-321), y sus 125 hallazgos también se muestran en la Tabla 1. Se observó *sliding* en la ecografía en todos los pacientes, lo que excluyó la presencia de neumotórax.

Se evaluó el grado de coincidencia entre los diagnósticos obtenidos mediante POCUS y radiografía. Los diagnósticos coincidieron en 70 pacientes. Los diagnósticos difirieron en los 30 pacientes restantes; en 20 casos la ecografía detectó patología no identificada en la radiografía, y en 10 casos la radiografía informó menos patología que la ecografía. Las discrepancias incluyeron 17 pacientes con atelectasias, 11 con derrame pleural (uno de ellos descrito como mínimo) y dos con edema pulmonar.

Los diagnósticos establecidos por el intensivista se muestran en la Tabla 2. Doce pacientes con edema pulmonar (57,1%) y otros 12 con neumonía (66,7%) tuvieron otros diagnósticos simultáneos.

Con los datos obtenidos, la ecografía mostró una sensibilidad del 85% (IC95%: 75-91,5) y una especificidad del 100% (IC95%: 79-100). El valor predictivo positivo fue100% (94-100), mientras que el valor predictivo negativo fue 55% (IC95%: 32-74).

**Tabla 2:** Diagnóstico final del intensivista

Diagnóstico del intensivista			
Principal	n	Otros	n (%)
Sin hallazgos de patología	29	-	
Edema pulmonar	21	atelectasia	4 (19,0)
	simple: 9 (42,9)	derrame pleural	8 (38,1)
Atelectasia	31	derrame pleural	1 (3,2)
Neumonía	18	edema pulmonar	3 (16,7)
	simple: 6 (33,3)	atelectasia	3 (16,7)
		derrame pleural	6 (33,3)
Derrame pleural simple	1	-	

## DISCUSIÓN

Nuestro estudio muestra que la POCUS, incluso cuando se utiliza con un protocolo abreviado, puede ser una herramienta más sensible que la radiografía portátil en la detección de patología pulmonar, respaldando hallazgos previos publicados tanto antes^4^ como después de la pandemia de COVID-19^14^^-^^16^. Este hecho sugiere que la POCUS puede desempeñar un papel esencial en el manejo del paciente crítico.

La capacidad de la ecografía para detectar patología, incluso subclínica, en un 20% de los pacientes cuya radiografía fue normal, permite la aplicación precoz de medidas terapéuticas para evitar la progresión de la enfermedad, tales como fisioterapia respiratoria agresiva, que revierta las atelectasias. Estudios recientes han respaldado esta utilidad, además de destacar un ahorro significativo en costes^9^^,^^17^^,^^18^.

El uso de un protocolo abreviado de POCUS pulmonar nos ha permitido reducir los tiempos de exploración descritos en la literatura, manteniendo una capacidad diagnóstica superior a la radiografía de tórax. Este enfoque abreviado, utilizado por operadores experimentados, ha acortado significativamente el tiempo medio de exploración ecográfica, pasando de los descritos previamente (14,8 minutos; DE=6,9) a tiempos muy inferiores (5,2 minutos). Estos tiempos, a su vez son muy inferiores a los tiempos medios descritos para radiografía de tórax (44,2 minutos; DE=21,4)^18^. Nuestro estudio sugiere que el uso de la ecografía en el cuidado diario del paciente crítico sería capaz de acortar el tiempo necesario para un diagnóstico precoz y, al mismo tiempo, reducir tanto el número de radiografías^10^^,^^11^ y la exposición a la radiación^19^ como los riesgos de traslados y movilizaciones.

Este estudio presenta dos limitaciones principales. En primer lugar, el uso de tan solo cuatro puntos en cada pulmón puede haber hecho que no detectáramos patología pulmonar, aunque la limitación del área de examen estaba justificada por la condición clínica del paciente^20^ y por el objeto del estudio de comprobar los resultados de una exploración abreviada, comparada con la radiología estándar de tórax. Tampoco el uso de protocolos más exhaustivos para el estudio de patología pulmonar ha demostrado ventajas claras^21^. La segunda limitación es que la ecografía es una exploración que depende de la persona que la ejecuta (operador) y que con los datos obtenidos debe emitir un diagnóstico.

Podemos usar la ecografía para evaluar el parénquima pulmonar, teniendo en cuenta la dificultad para descartar anomalías pulmonares que no alcancen la pleura, y para evaluar el espacio pleural. Ambas evaluaciones se pueden realizar con bastante fiabilidad tras una curva de aprendizaje relativamente breve y significativamente más corta que en otras técnicas ecográficas, aunque todavía requiere un entrenamiento adecuado centrado en la comprensión de la semiótica pulmonar de la ecografía y el correcto manejo clínico. Como toda prueba operador-dependiente, puede mostrar diferencias entre operadores. En nuestro estudio, todos los operadores habían realizado al menos 40 exploraciones previas supervisados por una persona experta en ecografía pulmonar. Se ha descrito que la exploración ecográfica pulmonar en pacientes críticamente enfermos requiere un programa de capacitación breve y fácil implementación basado en veinticinco exámenes de ultrasonido supervisados por personal médico con experiencia en ultrasonido pulmonar a pie de cama^22^.

A pesar de estas limitaciones, los datos obtenidos sugieren que la ecografía pulmonar tiene el potencial de reemplazar a la radiografía de tórax rutinaria en pacientes críticos. Aunque algunos trabajos sugieren la necesidad de utilizar ambas técnicas de manera complementaria^23^, nuestros resultados sugieren mejor capacidad diagnóstica de la ecografía en términos de sensibilidad y detección de patología.

Pensamos que la POCUS pulmonar representa una revolución en el manejo del paciente crítico debido a sus ventajas operativas y su ausencia de riesgo. Puede considerarse como un examen alternativo para la exploración del tórax en la UCI, proporcionando un diagnóstico rápido con buena reproducibilidad y alta viabilidad, especialmente en el derrame pleural y las atelectasias, patologías comunes en pacientes ingresados en UCI. Nuestro estudio sugiere que la POCUS pulmonar, utilizando un protocolo abreviado, puede ser una alternativa viable a la radiografía de tórax en la evaluación inicial y el seguimiento de la patología pulmonar en pacientes críticos. Estos hallazgos respaldan la utilidad clínica de la ecografía en la UCI y plantean interrogantes sobre la necesidad de radiografías rutinarias en este entorno.

En conclusión, la POCUS pulmonar abreviada al ingreso en la UCI no pasó por alto ninguna anomalía importante descrita posteriormente en la radiografía de tórax y mostró una concordancia moderada con la radiografía para el diagnóstico de edema pulmonar, consolidación alveolar y derrame pleural, principalmente debido a que detectó un mayor número de anomalías.

Se necesitan estudios adicionales para confirmar estos resultados y establecer pautas claras sobre el uso óptimo de la POCUS pulmonar en la UCI. Sin embargo, nuestros hallazgos respaldan la tendencia hacia una mayor integración de esta técnica en la práctica clínica diaria, lo que podría mejorar significativamente la calidad de la atención y el manejo de los pacientes críticos. 

## Data Availability

Se encuentran disponibles bajo petición al autor de correspondencia.
